# 

**Published:** 1865-04

**Authors:** 


### CONTENTS.

### ORIGINAL CONTRIBUTIONS.

ART. XII. PROTECTION FROM SMALL-POX BY MEANS OF VACCINATION AND RE-VACCINATION. By A. N. BELL, J. P. LOINES, H. D. BUCKLEY, A. NEBINGER, and JAMES F. HIBBARD, .......... 193

ART. XIII. ENLARGEMENT OF THE PROSTATE. By F. R. PAYNE, M.D., of Marshall, Illinois, .......... 205

ART. XIV. CEREBRO-SPINAL MENINGITIS—SPOTTED FEVER, &C. The Summary of a Clinic in the Medical Wards of the Mercy Hospital. April, 1865. By N. S. DAVIS, M.D., Prof. of Prac. and Clin. Med., 208

### FOREIGN CORRESPONDENCE.

LETTER FROM LIVERPOOL. By J., .......... 214

### PROCEEDINGS OF SOCIETIES.

Proceedings of the DeWitt County Medical Society. Reported by C. GOODBRAKE, M.D., Secretary, .......... 220

### SELECTIONS.

On a Case of Apoplexy of the Spinal Gray Substance, attended with Convulsions, with Pathological Examinations and Remarks. By M. Gonzalez Echeverria, A.M., .......... 221

On Some Points having Reference to the Differential Diagnosis of Fibrous Tumors of the Uterus. By Dr. C. H. F. Routh, Physician to the Samaritan Hospital for Women and Children, .......... 229

On the Treatment of Fibrous Tumors of the Uterus. By C. H. F. Routh, Physician to the Samaritan Hospital for Women and Children, .......... 236

### BOOK NOTICES.

Ninth Biennial Report of the Officers of the Illinois State Hospital for the Insane, .......... 243

Lectures on Surgical Pathology, delivered at the Royal College of Surgeons of England. By James Paget, F.R.S., Surgeon Extraordinary to her Majesty the Queen, &c., &c., .......... 243

The Pharmaceutists' and Druggists' Practical Receipt-Book, with a Glossary of Medical Terms, and Copious Index. By Thomas F. Branston, 243

History of a Case of Partial Reconstruction of the Face. By Gurdon Buck, M.D., Surgeon to the New York Hospital, &c., .......... 243

### EDITORIAL.

Vaccinate Report, .......... 247
Summer Medical Instruction, .......... 247
Obituary.—David Rutter, M.D., .......... 247
Epidemic of Plague in St. Petersburg, Russia, .......... 249
American Medical Association, .......... 252
Œsophagism, .......... 253
Pennsylvania Hospital, .......... 253
Citric Acid in Diabetes, .......... 254
Sour Milk to Irritable Ulcers, .......... 246

